# Effect of HM-Exos on the migration and inflammatory response of LPS-exposed dental pulp stem cells

**DOI:** 10.1186/s12903-023-02796-4

**Published:** 2023-02-14

**Authors:** Ehsaneh Azaryan, Samira Karbasi, Mansoore Saharkhiz, Mohammad Yahya Hanafi-Bojd, Asghar Zarban, Fariba Emadian Razavi, Mohsen Naseri

**Affiliations:** 1 Student Research Committee, Birjand University of Medical Sciences, Birjand, Iran; 2 Cellular and Molecular Research Center, Department of Molecular Medicine, Birjand University of Medical Sciences, Birjand, Iran; 3 Department of Molecular Medicine, School of Medicine, Cardiovascular Diseases Research Center, Birjand University of Medical Sciences, Birjand, Iran; 4 Department of Immunology, Faculty of Medicine, Birjand University of Medical Sciences, Birjand, Iran; 5 Cellular and Molecular Research Center, Birjand University of Medical Sciences, Birjand, Iran; 6 Department of Pharmaceutics and Pharmaceutical Nanotechnology, School of Pharmacy, Birjand University of Medical Sciences, Birjand, Iran; 7 Cardiovascular Diseases Research Center, Birjand University of Medical Sciences, Birjand, Iran; 8 Clinical Biochemistry Department, Faculty of Medicine, Birjand University of Medical Sciences, Birjand, Iran; 9 Dental Research Center, Faculty of Dentistry, Birjand University of Medical Sciences, Birjand, Iran

**Keywords:** Dental pulp stem cells, Exosomes, Inflammation, Lipopolysaccharide

## Abstract

**Aim:**

The purpose of this study was to investigate the effects of human milk exosomes (HM-Exos) on the viability, migration, and inflammatory responses of lipopolysaccharide (LPS)**-**exposed human dental pulp stem cells (HDPSCs) in vitro.

**Methods:**

HM-Exos were isolated, and dynamic light scattering (DLS), scanning electron microscopy (SEM), and transmission electron microscopy (TEM) were used to analyze their physical properties (size and shape). To construct an in vitro inflammation model, HDPSCs were exposed to LPS. The MTT test and migration assay were used to investigate the effect of HM-Exos on cell proliferation and migration, and the quantitative polymerase chain reaction (qPCR) was used to assess the expression of inflammatory genes in HDPSCs. Data were analyzed using a one-way analysis of variance (ANOVA) with Tukey's post-test.

**Results:**

DLS measurement revealed that HM-Exos were 116.8 ± 3.6 nm in diameter. The SEM and TEM images revealed spherical shapes with diameters of 97.2 ± 34.6 nm. According to the results of the cell viability assay, the nontoxic concentration of HM-Exos (200 µg/ml) was chosen for the subsequent investigations. The migration assay results showed that HM-Exos improved the potential of LPS-exposed HDPSCs to migrate. The qPCR results indicated that HM-Exos significantly reduced the expression of inflammatory cytokines such as TNF-α, IL-1β, and IL-6 in HDPSCs after LPS stimulation.

**Conclusions:**

HM-Exos increased LPS-exposed HDPSCs migration and proliferation and reduced gene expression of inflammatory cytokines. They may be a viable candidate for pulpitis therapy.

**Supplementary Information:**

The online version contains supplementary material available at 10.1186/s12903-023-02796-4.

## Highlights

- HM-Exos were successfully isolated using the exosome extraction kit.
- The size and shape of HM-Exos were analyzed using DLS, SEM, and TEM.
- HM-Exos improved the migration potential of LPS-exposed HDPSCs.
- HM-Exos reduced the expression of inflammatory cytokines such as TNF-α, IL-1β, and IL-6 in LPS-exposed HDPSCs.

**Supplementary Information:**

The online version contains supplementary material available at 10.1186/s12903-023-02796-4.

## Introduction

Tooth decay, dental trauma, tooth erosion, and other dental problems can all stimulate inflammation in the dental pulp tissue, leading to pulp necrosis and even periapical periodontitis [1]. One of the prevalent dental conditions known as pulpitis is an opportunistic infection caused by oral bacteria, which cause tooth pulp inflammation [2]. Lipopolysaccharide (LPS) is a potent stimulator of pulpitis that has been found in inflamed pulp tissue, causing the release of inflammatory cytokines including TNF-α, IL-1β, and IL-6 [3–5]. Upregulation of these pro-inflammatory mediators is related to a decrease in odontoblastic markers and the stimulation of pulp necrosis [6, 7].

When the pulp is damaged, it requires vital pulp therapy to keep it alive and functional. A suitable pulp capping substance should protect the pulp from infection and create a favorable biological environment for the regeneration of dental pulp tissue [8]. Depending on the degree of inflammation, indirect pulp therapy, direct pulp capping, and pulpotomy can be done to protect the vitality of dental pulp. The most regularly utilized pulp caps have limited anti-inflammatory capabilities. On the other hand, sodium hypochlorite and normal saline are the two most frequently utilized washing solutions in pulpotomies. However, normal saline has no anti-inflammatory qualities, while sodium hypochlorite in high concentrations damages the pulp tissue [6]. Anti-inflammatory drugs can therefore be used to treat pulpotomy and reduce pulp inflammation.

Exosomes are extracellular vesicles released by various cell types and have a diameter of 30–150 nm. They contain various types of components obtained from source cells, such as proteins, miRNAs, RNA, and DNA, which conduct a significant role in cell-to-cell interaction [9]. They are involved in regulating important biological processes such as the immune response and inflammation, collagen production, tissue regeneration, blood coagulation, and angiogenesis [9–11]. Moreover, exosomes are being employed more often as a component in wound healing and scar-free procedures. Because they are naturally generated, they can regulate inflammatory responses and encourage cell migration and proliferation [11].

Breast milk includes a wide range of components, including immune competent cells, milk fat globules (MFG), soluble proteins such as IgA, cytokines, and antimicrobial peptides [12]. Exosomes are found in abundance in breast milk [13]. Milk exosomes are membranous nanovesicles that are 30–100 nm in size and play an important role in intercellular communication, primarily through their mRNAs, microRNAs, and proteins [14]. Milk-derived exosomes have been shown to effectively reduce the inflammatory response induced by LPS. Furthermore, the protective properties of milk exosomes are quite fascinating and imply that these nanovesicles might be useful in the treatment of numerous inflammation-related disorders [15].

Mesenchymal stem cells (MSCs) are multipotent stem cells found in a variety of adult tissues, including bone marrow, adipose tissue, and tooth pulp. These cells are distinguished by their ability to self-renew and multi-differentiate [16–18]. MSCs have a high paracrine influence on the body. Considering the role of exosomes in cellular communication, it is suggested that these molecules play a role in the paracrine actions of MSCs [19]. The first stem cells derived from adult human dental pulp were dental pulp stem cells (DPSCs). These cells are separated from the third molar and have a high rate of proliferation and self-renewal [20]. Because of their tremendous potential in tissue repair/regeneration and immune response regulation, they have been indicated as a prospective choice for cell-based therapeutic applications. Therefore, it has been presented as a promising therapy for inflammatory and damaging disorders such as pulpitis [8, 16, 21].

As a result, we constructed a model of LPS-induced inflammation in human dental pulp stem cells (HDPSCs), and the effects of human milk exosomes (HM-Exos) on the proliferation, migration, and inflammatory response of LPS-induced HDPSCs were investigated.

## Materials and methods

Dulbecco’s modified Eagle’s medium (DMEM) and fetal bovine serum (FBS) were supplied by Gibco, Grand Island. Penicillin plus streptomycin solution, Escherichia coli LPS powder, and 3-(4,5- dimethylthiazol-2-yl)-2,5-diphenyltetrazolium bromide (MTT) were obtained from Sigma-Aldrich (St. Louis, MO, USA). Exosome extraction kit (Exocib) was supplied by Cibbiotech Co (Tehran, Iran). Dimethyl sulfoxide (DMSO) provided from Merck Chemical Co.

### HM-Exos isolation

For this cross-sectional study, 350 breastfeeding mothers aged 20–35 were recruited from four healthcare centers in southern Khorasan, Birjand, Iran. A cluster random sampling method was used to select participants. The sample size for this study was determined according to 80% power and the following formula: n = [(Z1−α/2)2−S2]/d2 which SD = 5.2, d = 0.504, and α = 0.1. According to this assessment, 294 participants were needed; therefore, owing to the availability of data and to account for every possible exclusion, 350 participants were added. Birjand University of Medical Sciences Ethics Committee (ethical number: IR.BUMS.REC.1399.281) approved the study. Before recruitment, subjects provided written informed consent. All participants had infants aged 1–6 months, with no history of chronic diseases or medication use in the previous six months. At the start of the day, each mother was asked to provide two samples of breast milk in 20 ml volumes expressed from primary breastfeeding. Breast milk is centrifuged after collection to remove fat and debris. The upper-fat layer is removed by centrifuging milk at 300 g for 45 min at 4 °C. The supernatants were centrifuged at 2000*g* for 1 h at 4 °C to further remove deposited cells and then at 12,000 g for 1 h, and 14,000 g for 2 h at 4 °C to pellet the vesicles [22, 23]. Then exosomes are extracted from the remaining milk by the EXOCIB kit as directed by the manufacturer. A BCA protein assay kit (Pars Tous, Tehran, Iran) was used to determine the total protein content of exosomes.

### Exosome characterization

DLS (NanoBrook 90Plus) was used to evaluate the size of Exos, and to further analyze the size and morphology of Exos, SEM (FEI Quanta 200) and TEM (Philips EM 208S) were employed.

### HDPSC isolation and culture

The HDPSCs were isolated using a technique reported in our prior publication [21]. In brief, healthy third molars (wisdom teeth) of patients aged 20 to 25 were collected after obtaining the consent of the patients at the Dental Center of Imam Reza Hospital in Birjand (Iran), following the guidelines of the Birjand University of Medical Sciences ethics committee (Ethical Number: IR.BUMS.REC.1399.090). The pulp tissues were removed from the teeth and then soaked in PBS containing 100 U/mL type I collagenase enzyme for 1 h at 37 °C. The extracted cells from the tooth pulp were cultured in DMEM, complemented by 10% FBS and 1% antibiotic (penicillin–streptomycin), at 37 °C in 5% CO2 and a humidified atmosphere (Additional file 1). Cell passage numbers from 3 to 5 were used for the following experiments.

### LPS induction of HDPSCs and treatment of HDPSCs with various LPS concentrations

According to published methods [1, 24], Escherichia coli LPS powder was dissolved in distilled water to make the 1 mg/ml LPS stock solution. After this, serial dilution was used to prepare a series of LPS concentrations, including 0.5, 1, 2, 4, and 8 μg/mL. HDPSCs were planted in a 96-well plate (SPL) at a density of 1.5 $\times$ 10^4^ cells per well and incubated in culture media containing various concentrations of LPS for 24 h. These LPS-induced HDPSCs were called inflammatory human dental pulp stem cells (iHDPSCs). Then 20 µl of MTT solution (2 mg/ml in PBS) was added to each well, and the plate underwent incubation for 4 h at 37 °C incubator in the dark. Afterward, the supernatant was eliminated, 100 µl DMSO was added to each well, and the optical absorption of the samples was measured at 570 and 630 nm using a spectrophotometer (Biotek Epoch, Winooski,VT).

### Cell viability assay of iHDPSCs with various HM-Exo concentrations

For the cell viability test, HDPSCs were planted in a 96-well plate at a density of 1.5 $\times$ 10^4^ cells per well. After 24 h of LPS induction (1 µg/ml), the medium was changed to complete culture medium supplemented with different doses of HM-Exo for 24 h at 37 °C. Then 20 µl of MTT solution was added to each well, and the plate underwent incubation for 4 h at 37 °C in the dark. Afterward, the supernatant was eliminated, 100 µl DMSO was added to each well, and the optical absorption of the samples was measured at 570 and 630 nm using a spectrophotometer.

### Cell migration assay

A scratch test was used to evaluate the migration potential of iHDPSCs. HDPSCs were planted into six‐well plates. After 24 h of LPS induction (1 µg/ml), the medium was changed to complete culture medium, and a straight scratch was formed with a 200 μl tip. Microscopy was used to observe cell migration at 0, and 24 h after treatment with 200 µg/ml of HM-Exos. The lesion's boundary areas were evaluated and photographed using an inverted microscope (ZEISS Axiovert 200, Zeiss, Germany). Cellular migration was evaluated by measuring the ratio between the reduced open space after 24 h and the open space at 0 h.

### Quantitative real-time polymerase chain reaction (q-PCR)

HDPSCs were planted into the plates at a density of 3 $\times$ 10^5^ cells. After 24 h of LPS induction (1 µg/ml), the mRNA expression levels of TNF-α, IL-1β, and IL-6 were evaluated using the quantitative real-time polymerase chain reaction and the GAPDH gene was used as an endogenous control. Isolation of RNA and cDNA synthesis were performed using the Pars Tous kit (Tehran, Iran) according to the manufacturer’s instructions, and real-time was done using the SYBR Green assay. The expression levels of target genes were calculated using the 2^−ΔΔCt^ method. The sequences of primers used are shown in Table 1.

**Table 1:** Sequences of primers used for real-time PCR

Name	Forward	Reverse
TNF alpha	AGGCGGTGCTTGTTCCTCAG	GGCTACAGGCTTGTCACTCG
IL-1β	TCCAGGGACAGGATATGGAG	TCTTTCAACACGCAGGACAG
IL-6	AGACTTGCCTGGTGAAAATCA	GCTCTGGCTTGTTCCTCACT
GAPDH	CGAACCTCTCTGCTCCTCCTGTTCG	CATGGTGTCTGAGCGATGTGG

### Statistical analysis

The data were presented as mean standard deviation. The statistical analysis was carried out using GraphPad Prism 9 software (GraphPad Software, Inc, La Jolla, CA). A one-way analysis of variance (ANOVA) with Tukey's posthoc multiple comparison test was employed to assess statistical significance for noticing significant differences between study groups, with *p* < 0.05 as the statistical significance value. Mean and standard deviation (SD) were determined (Additional file 1).

## Result

### HM-Exo isolation and characterization

**Fig. 1:**
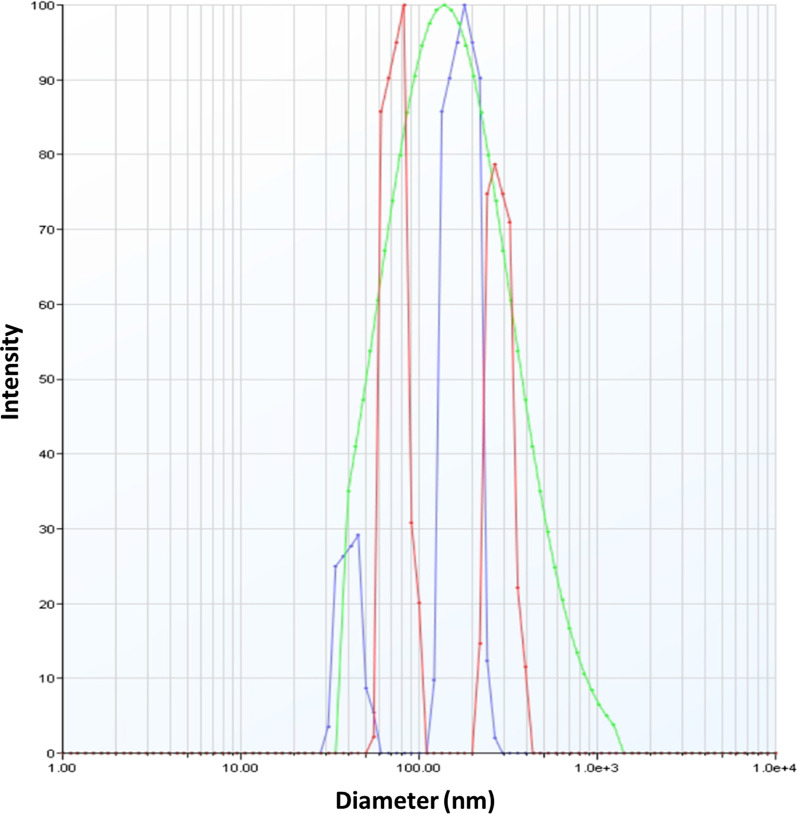
DLS measured the particle size distribution (by intensity) of isolated HM-Exo

**Fig. 2:**
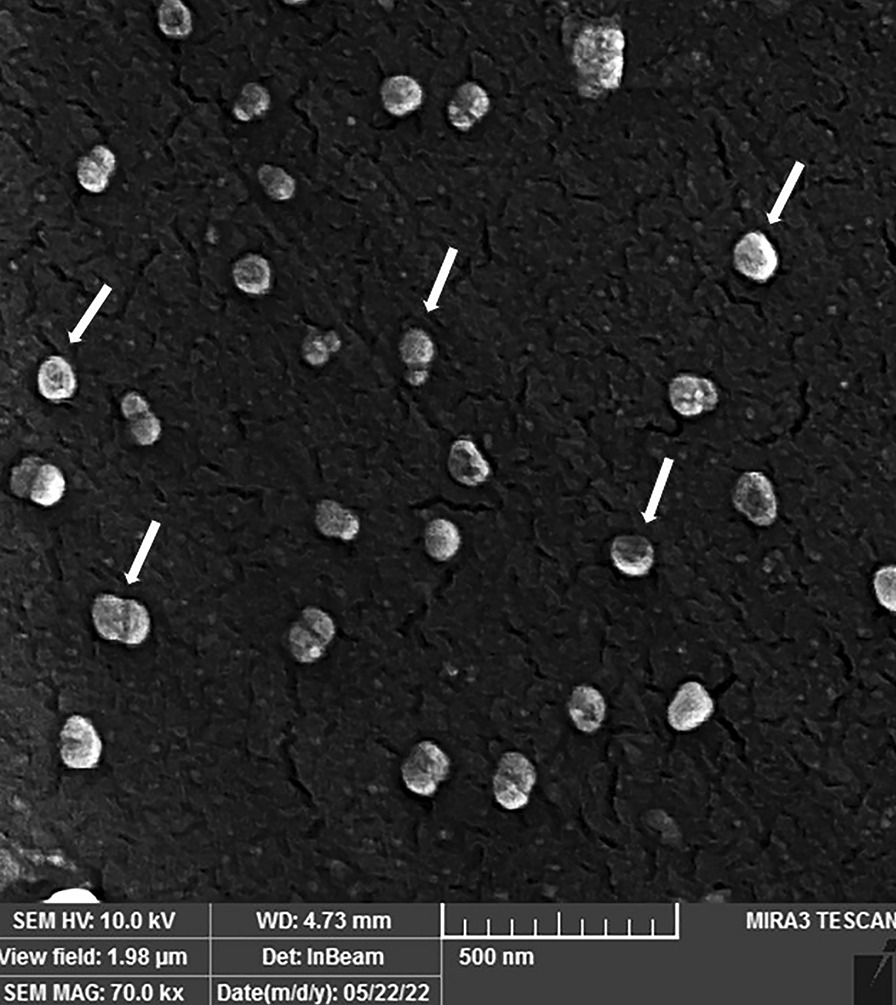
SEM micrograph of HM-Exo demonstrates small nanovesicles

**Fig. 3:**
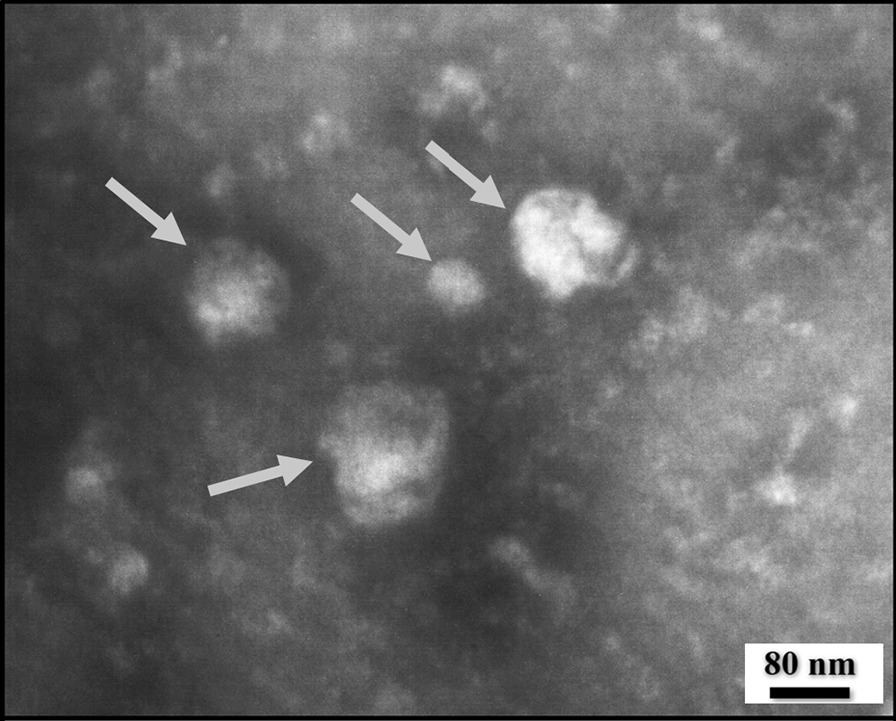
TEM micrograph of HM-Exo demonstrates small nanovesicles

According to DLS findings, HM-Exos were 116.8 ± 3.6 nm in diameter (Fig. 1). The SEM and TEM observations of Exos revealed that they were spherical and had a size of 90 nm or more (Figs. 2 and 3).

### Effect of LPS powder on HDPSCs viability

**Fig. 4:**
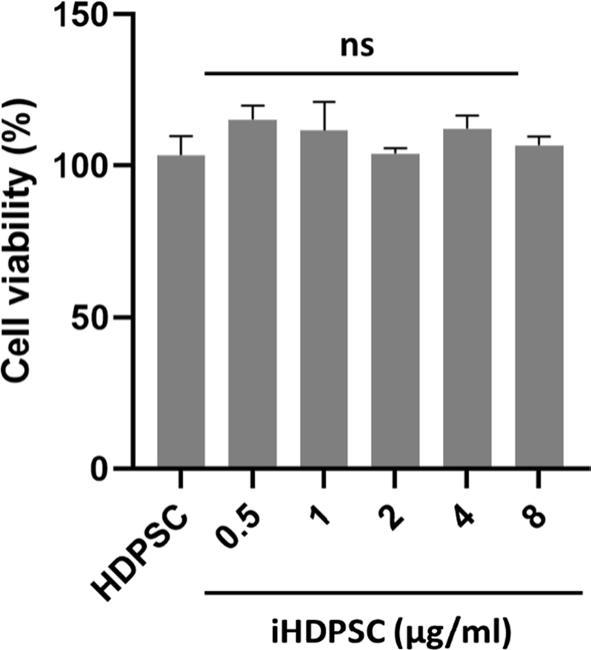
Viability assay for HDPSCs (control) and iHDPSCs incubated with LPS on 24 h. ns; nonsignificant

The effect of LPS powder (0.5–8 µg/ml) on the cell viability of HDPSCs is shown in Fig. 4. After 24 h, the viability of cells treated with LPS was not significantly different compared to the control group (*p* > 0.05). 1 µg/ml of HM-Exos was chosen for the following investigations.

### Effect of HM-Exos on iHDPSCs viability

**Fig. 5:**
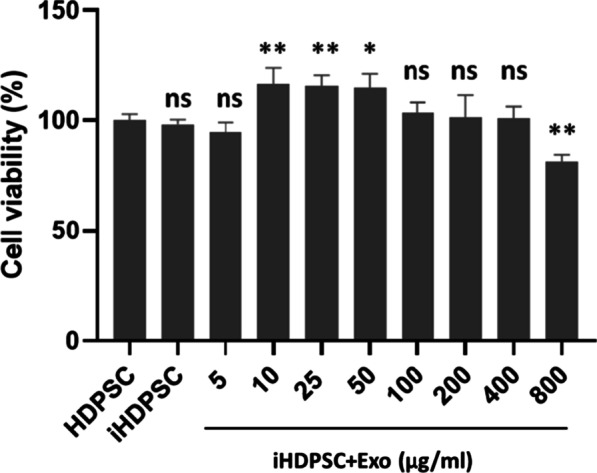
Viability assay for HDPSCs (control) and iHDPSCs incubated with HM-Exos on 24 h. Significant differences compared to control were indicated: *p < 0.05; **p < 0.01. ns; nonsignificant

The effect of LPS powder (1 µg/ml) and different doses of HM-Exos on the cell viability of iHDPSCs is shown in Fig. 5. After 24 h, the viability of HM-Exos-treated iHDPSCs increased significantly at doses of 10 and 25 (*p* < 0.01) and 50 µg/ml (*p* < 0.05). In comparison to the control group, no statistically significant difference in cell toxicity or proliferation was seen at 5 and 100–400 µg/ml doses (*p* > 0.05). Furthermore, at 800 µg/ml, cell viability was reduced (*p* < 0.01). Based on the findings of the cell viability assay, the concentration of 200 µg/ml was chosen for the following investigations.

### The effect of HM-Exos on iHDPSCs migration

**Fig. 6:**
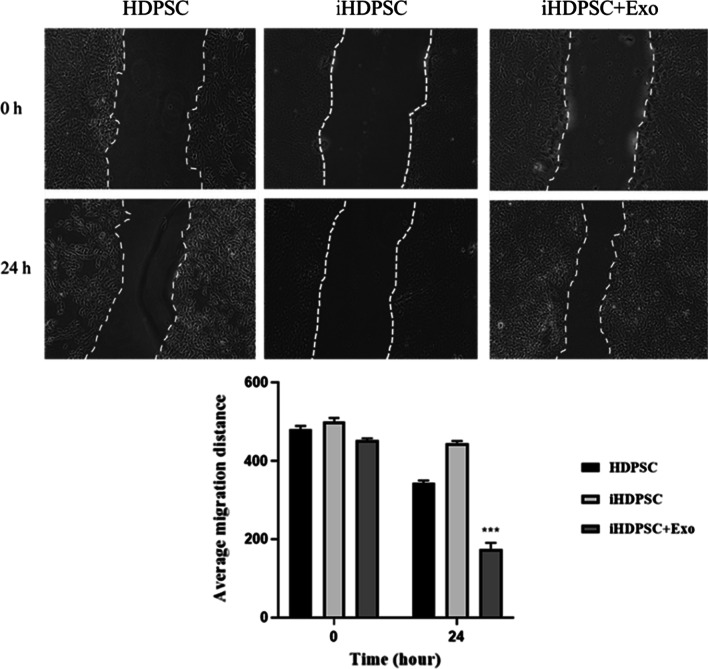
The effect of HM-Exos on the migration of iHDPSCs. Significant differences compared to control were indicated: ***p < 0.001

To investigate the effect of HM-Exos on the migration capacities of iHDPSCs, the scratch test was done (Fig. 6). The results indicated that the concentration of 200 μg/ml of HM-Exos improved the migratory abilities of iHDPSCs  compared to HDPSC and iHDPSC groups (*P* < 0.001).

### The effect of LPS powder and HM-Exos on inflammatory cytokines expression

**Fig. 7:**
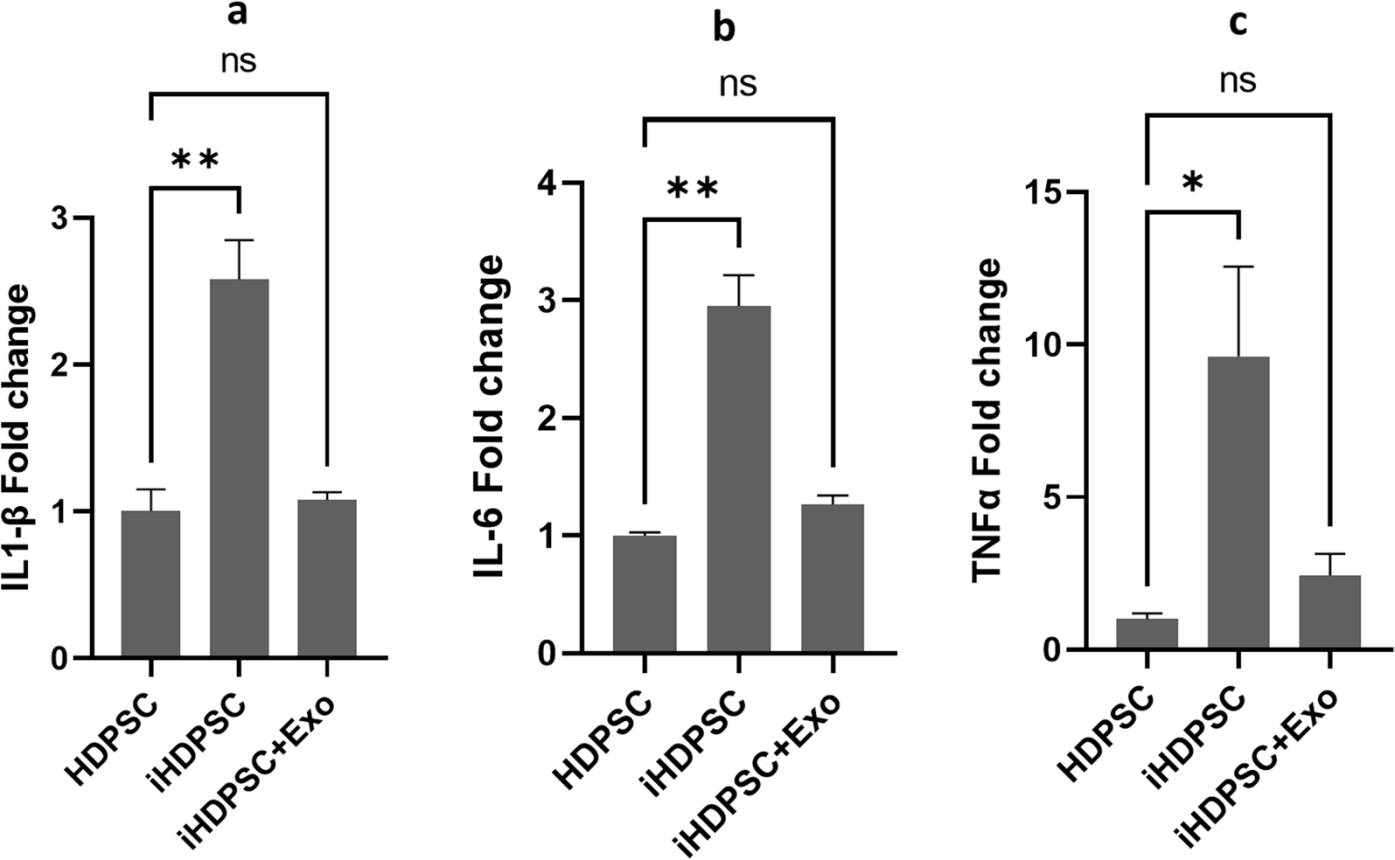
Comparison of the genes expression levels: **a** IL1-β, **b** IL-6, and **c** TNFα. *p < 0.05, **p < 0.01. ns; nonsignificant

To investigate the inhibitory impact of HM-Exos on mRNA expression of inflammatory cytokines in iHDPSCs, q-PCR was used (Fig. 7). LPS significantly increased mRNA expression of IL1-β, IL-6, and TNFα (*p* < 0.01, *p* < 0.01, *p* < 0.05) respectively compared to the HDPSC group. HM-Exos significantly reduced LPS-induced expression of IL1-β, IL-6 (*P* < 0.01), and TNFα (*P* < 0.05) compared to the iHDPSC group.

## Discussion

The expression of inflammatory cytokines can be stimulated in DPSCs by LPS, a significant component of the bacterial outer membrane [5]. Immunosuppressive cytokines like transforming growth factor beta (TGF-β) and beneficial miRNAs such as miRNA-148 are abundant in mammalian milk exosomes [25]. Due to the presence of immune-regulatory miRNAs, milk-derived extracellular vesicles can affect the immune system of milk recipients [15]. However, the role of HM-Exos in pulpitis therapy remained unclear. We isolated human milk exosomes. Using electron microscopy and DLS, we discovered that the vesicles had an exo-like form and size. We have demonstrated that HM-Exos had a positive effect on proliferation, migration, and decrease of inflammatory cytokines in an in vitro LPS model of pulpal inflammation. According to the result of the MTT assay, LPS (0.5–8 μg/ml) treatment of HDPSCs for 24 h had no significant effect on cell viability. He et al. revealed that LPS had no significant effect on DPSCs numbers up to 10 μg/ml for 1, 3, 5, and 7 days, as depicted in our study [26]. Previous studies have shown that the most effective approach for simulating the inflammatory microenvironment in vitro is to treat HDPSCs with LPS at a concentration of 1 µg/ml. Therefore, we used 1 μg/ml LPS to construct an in vitro inflammatory model [1, 24]. When the pulp tissue becomes inflamed, DPSCs proliferate and migrate to the site of lesion, differentiate into odontoblast-like cells, and secrete extracellular matrix molecules to form dentin and preserve pulp vitality. Therefore, it is essential to regulate DPSC responses to dental pulp inflammation to preserve pulp vitality and produce restored dentin. As a result of these inflammatory conditions, tooth repair necessitates the involvement of several physiological DPSC processes, including cell proliferation, migration, and differentiation [1]. Our results showed that HM-Exos had a positive impact on both the proliferation and migration of iHDPSCs. Although LPS alone had no effect on the migration of HDPSCs, HM-Exos could accelerate the migration of iHDPSCs.

In this investigation, LPS induced an inflammatory condition in DPSCs by increasing the expression of TNF-α, IL-1β, and IL-6. In addition, we indicated that the level of inflammatory cytokines reduced after treatment with HM-Exos.

Pulpitis is a common inflammation of tooth pulp tissue, and oral microbes are implicated in this opportunistic infection. According to research, various parameters associated with host reaction play an important role in pulpitis. Among these components are immune system inflammatory mediators such as cytokines and chemokines, which contribute to pulpal defense mechanisms [2]

In this regard, the results of a reversible pulpitis model showed that IL-1β, IL- 6, and TNF-α gene expressions were elevated in LPS-exposed inflamed pulp tissues [27]. Recent studies on the cellular and molecular basis, inflammatory processes, pulp repair, and the emergence of new drug strategies such as drug delivery systems and tissue engineering have opened up numerous ways for the progress of infectious and inflammatory pulp therapeutic strategies [28]. Jung et al. revealed that simvastatin inhibits the expression of inflammatory cytokines, cell adhesion molecules, and NF-kβ transcription factors induced by LPS in human dental pulp cells [29]. Li et al. investigated that epigallocatechin gallate (EGCG) significantly reduced expression of inflammatory cytokines like TNF-α, IL-1β and IL-6 in dental pulp stem cells after LPS stimulation, and also reduced inflammation of inflamed rat pulp tissue [6]. Chen et al. demonstrated that 4-Methylumbelliferone decreased inflammatory cytokines and promoted migration and odontogenic differentiation of LPS-induced DPSCs [1]. According to several studies, exosomes have the potential to regulate immunity in inflammatory diseases. For example, exosomes from IL-10-treated dendritic cells were found to be effective in both suppressing the onset of murine collagen-stimulated arthritis and reducing the symptoms of established arthritis [30]. According to one study, Exos-derived MSCs can reduce osteoarthritic symptoms in an inflammatory model of osteoarthritis [31]. Another study discovered that bone marrow mesenchymal stem cell-derived exosomes boosted M2 macrophages, decreased inflammatory cytokines, and induced the secretion of anti-inflammatory cytokines [32]. Another study found that human breast milk exosomes reduced hypoxia and LPS-stimulated inflammation in damaged intestinal organoids, as well as the expression of IL-6 under injury conditions [13]. Reif et al. indicated that in a colitis murine model, human milk-derived exosomes reduced the expression of IL-6 and TNF-α compared with the untreated group [25]. Ahn et al. found that bovine milk exosomes inhibited the expression level of inflammatory cytokines such as IL-6 and TNF-α in LPS-induced RAW264.7 cells [11].

Exosome therapy is emerging as a viable treatment option for a variety of disorders, particularly inflammatory ones. The use of exosomes in clinical applications and disease therapy is attended by limitations such as the difficulty in identifying an effective separation and purification approach, the inadequate standardization of large-scale production processes, and target cell uptake capacity [33].

## Conclusion

In conclusion, the results showed that HM-Exos therapy could not only increase iHDPSC migration and proliferation but also reduce inflammatory cytokine gene expression. Although these findings are promising, more in vitro and in vivo studies must be conducted in the future to demonstrate the efficacy of HM-Exos in pulpitis therapy.

## Supplementary Information

**Additional file 1**. **Fig. 1.** HDPSC isolation and differentiation to osteogenic and adipogenic lineages. **Fig. 2.** Immunophenotypic characterization of DPSCs. **Table 1.** Mean and standard deviation (SD) values of cell viability in MTT assay. **Table 2.** Mean and standard deviation (SD) values of cell viability in MTT assay. **Table 3.** Mean and standard deviation (SD) values of relative gene expression in q-PCR assessment.

## Data Availability

All data obtained or assessed during this investigation are contained in this article as "Additional file 1" in the Statistical analysis part of the method portion.

## Abbreviations

MSC: Mesenchymal stem cell
HDPSC: Human dental pulp stem cell
HM-Exo: Human milk exosome
LPS: Lipopolysaccharide
IL-6: Interleukin-6
TNF-α: Tumor necrosis factor-alpha
IL-1β: Interleukin-1 beta
iHDPSC: Inflammatory human dental pulp stem cells
