# Supplementary material for: Effect of HM-Exos on the migration and inflammatory response of LPS-exposed dental pulp stem cells
Source: BMC Oral Health. 2023 Feb 14;23:95. doi: 10.1186/s12903-023-02796-4 (PMC9926843; doi:10.1186/s12903-023-02796-4)
Supplement: Supplementary file 1 — Additional file 1. Fig. 1. HDPSC isolation and differentiation to osteogenic and adipogenic lineages. Fig. 2. Immunophenotypic characterization of DPSCs. Table 1. Mean and standard deviation (SD) values of cell viability in MTT assay. Table 2. Mean and standard deviation (SD) values of cell viability in MTT assay. Table 3. Mean and standard deviation (SD) values of relative gene expression in q-PCR assessment. [file 12903_2023_2796_MOESM1_ESM.docx]

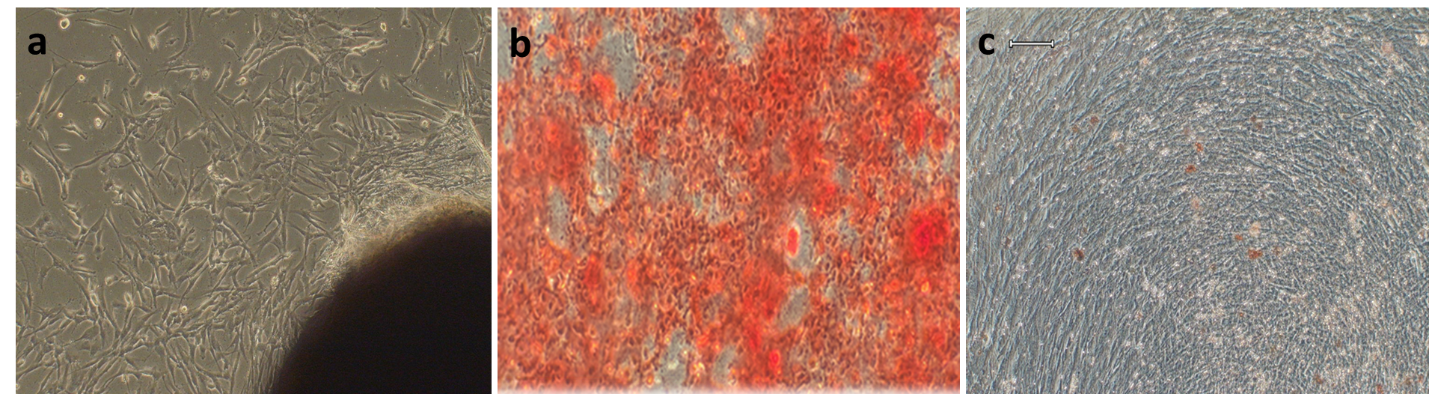


**Fig 1.** HDPSC isolation and differentiation to osteogenic and adipogenic lineages; **(a)** Migration of HDPSCs from pulp tissue and change morphologically into pure fibroblast-like, spindle-shaped cells after 3 passages. **(b)** Red cells with mineralized nodules after alizarin red staining on 28th day of osteo-differentiation. **(c)** Adipogenesis was confirmed by the red appearance of cytoplasmic lipids that were stained with the oil red reagent. HDPSCs; human dental pulp stem cells.


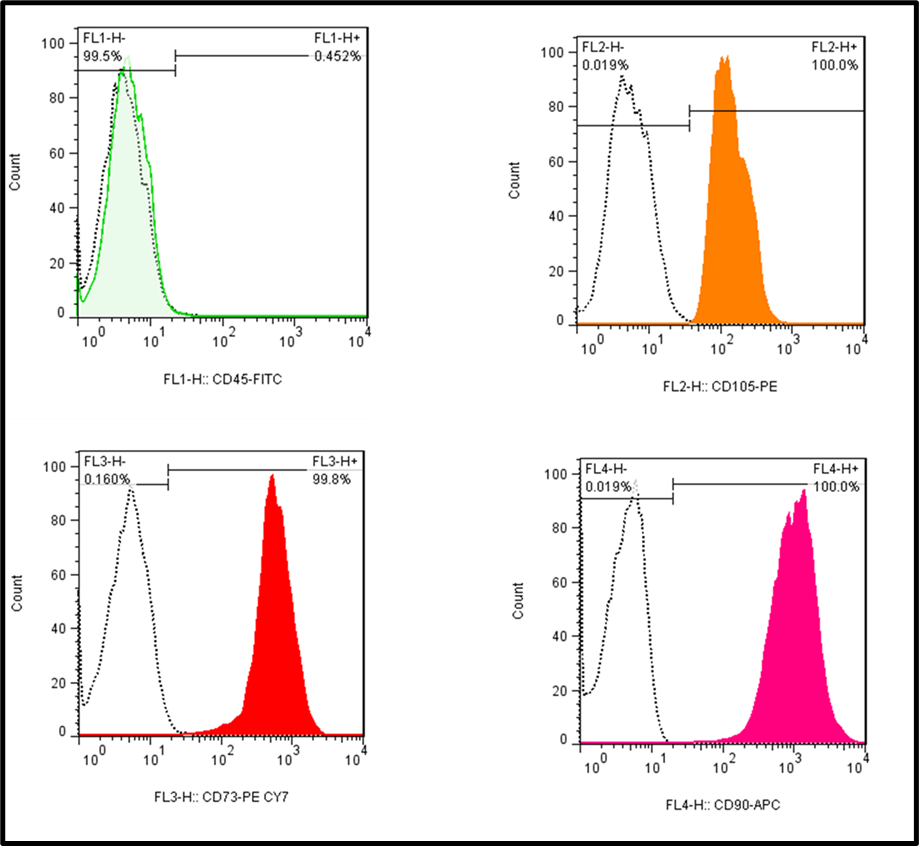


**Fig 2.** Immunophenotypic characterization of DPSCs. Flow cytometry analysis revealed that extracted cells were positive for specific mesenchymal markers CD90, CD73, and CD105, but negative for hematopoietic marker CD45.

**Table 1.** Mean and standard deviation (SD) values of cell viability in MTT assay. HDPSC; Human dental pulp stem cell (Control), iHDPSC; inflammatory HDPSC, HM-Exo; Human Milk exosome

| **SD** | **Mean** | **Concentration (µg/ml)** | **Groups** |
| --- | --- | --- | --- |
| **2.72** | **100.1** | **HDPSC** | **iHDPSC+Exo** |
| **2.18** | **98.15** | **iHDPSC** |  |
| **4.4** | **94.65** | **5** |  |
| **7.39** | **116.3** | **10** |  |
| **4.70** | **115.6** | **25** |  |
| **6.20** | **114.7** | **50** |  |
| **4.71** | **103.4** | **100** |  |
| **10.1** | **101.1** | **200** |  |
| **5.36** | **100.8** | **400** |  |
| **3.14** | **81.34** | **800** |  |

**Table 2.** Mean and standard deviation (SD) values of cell viability in MTT assay.

| **SD** | **Mean** | **Concentration (µg/ml)** | **Groups** |
| --- | --- | --- | --- |
| **6.19** | **103.4** | **HDPSC** | **iHDPSC** |
| **4.45** | **115.2** | **0.5** |  |
| **9.37** | **111.5** | **1** |  |
| **1.75** | **103.9** | **2** |  |
| **4.29** | **112.2** | **4** |  |
| **2.86** | **106.7** | **8** |  |

**Table 3.** Mean and standard deviation (SD) values of relative gene expression in q-PCR assessment.

| **SD** | **Mean** | **Groups** | **Targeted gene** |
| --- | --- | --- | --- |
| **0.14** | **1.005** | HDPSC | **IL1-β** |
| **0.26** | **2.582** | iHDPSC |  |
| **0.05** | **1.080** | iHDPSC+Exo |  |
| **0.02** | **0.999** | HDPSC | **IL6** |
| **0.26** | **2.952** | iHDPSC |  |
| **0.07** | **1.267** | iHDPSC+Exo |  |
| **0.19** | **1.004** | HDPSC | **TNFα** |
| **2.94** | **9.620** | iHDPSC |  |
| **0.71** | **2.433** | iHDPSC+Exo |  |
